# National and regional economic inequalities in first- and second-hand tobacco consumption among women of reproductive ages in Iran

**DOI:** 10.1186/s12889-023-17287-y

**Published:** 2023-12-18

**Authors:** Samira Arabi, Nader Jahanmehr, Maryam Khoramrooz

**Affiliations:** 1 School of Public Health, Tehran University of Medical Sciences, Tehran, Iran; 2 Health Economics, Management, and Policy Department, Virtual School of Medical Education & Management, Shahid Beheshti University of Medical Sciences, Tehran, Iran; 3 Prevention of Cardiovascular Disease Research Center, Shahid Beheshti University of Medical Sciences, Tehran, Iran; 4 Department of Health Management and Economics, School of Public Health, Hamadan University of Medical Sciences, Hamadan, Iran; 5 Modeling of Noncommunicable Diseases Research Center, Hamadan University of Medical Sciences, Hamadan, Iran

**Keywords:** Economic inequality, Socioeconomic factors, Tobacco consumption, Second-hand smoke, Women of reproductive ages, Iran

## Abstract

**Introduction:**

The epidemic of tobacco consumption is one of the major public health threats the world has been facing so far. This study was performed to investigate the economic inequalities in tobacco consumption among women of reproductive ages at national and regional levels in Iran.

**Methods:**

We used data from 10,339 women of reproductive ages (18–49 years) who participated in Iran’s 7th Non-Communicable Disease Risk Factor Surveillance (STEPS). Wagstaff normalized concentration index and decomposition method were applied to measure economic inequalities in first- and second-hand tobacco consumption and determine their corresponding contributory factors, respectively.

**Results:**

The prevalence of women’s first-hand tobacco consumption, and their exposure to second-hand smoke in the home, and workplace were 3.6%, 28.3%, and 8.4%, respectively. First- and second-hand tobacco consumption was significantly more concentrated among low-economic women. Exposure to home second-hand smoke, education, and economic status had the largest contributions to the measured inequality in first-hand tobacco consumption (48.9%, 38.9%, and 30.8%, respectively). The measured inequality in women’s secondhand smoke exposure at home was explained by their level of education (43.8%), economic status (30.3%), and residency in rural areas (18%), and at work by residency in rural areas (42.2%), economic status (38.8%), and level of education (32%). Our results also revealed diversity in the geographical distribution of inequalities in rural and urban areas and five regions of the country.

**Conclusion:**

The present study highlighted the need for more enforcement of tobacco control rules and increasing tobacco taxes as general measures. Furthermore, there is a need for gender-sensitive initiatives at national and regional levels to educate, support, and empower low-economic women and households for tobacco cessation, and complying with restrictive smoking rules.

## Introduction

The epidemic of tobacco consumption is one of the major public health threats the world has been facing so far. It is a major risk factor for chronic non-communicable diseases and one of the most important causes of morbidity, mortality, and disability worldwide [1–3]. Annually, 8 million people die in terms of tobacco smoking, and 1.2 million deaths occur in the world because of exposure to second-hand smoke [3]. It is estimated that by 2030, the number of fatalities attributable to tobacco will increase to 8.3 million per year [4]. The majority of fatalities occur in low- and middle-income countries [1, 3], where individuals are susceptible to the adverse effects of smoking due to poor health system performance and destitution [5]. Tobacco consumption incurs staggering economic costs to society via increasing medical costs and reducing productivity [6]. In most economies, the burden of healthcare costs resulting from tobacco consumption is more than the governments’ tax revenues from tobacco products [7]. The World Health Organization (WHO) has established the Tobacco Control Program since 1998. In 2003, the Framework Convention on Tobacco Control (FCTC) was adopted the by the 56th World Health Assembly [8]. Iran joined FCTC in 2005, and it was implemented in Iran since 2006 [9]. Within this framework, Iran has implemented some tobacco control policies, such as tobacco taxes, graphic warning labels on cigarette packaging, advertising ban on national television, radio and print media, and bans on smoking in public places [10, 11]. Although Iran had the best score among the countries of the Eastern Mediterranean region in 2017 [12], however, these policies were not implemented as strongly as needed. For example, taxes on tobacco products in Iran are < 25% of retail price [11], and the ban on smoking in public places is not sufficiently enforced [10]. Furthermore, a free-of-charge national tobacco cessation hotline was established in June 2021, and all costs of smoking cessation services in public health centers are covered [13], excluding the costs of drugs.

In Iran, 0.26% of the GDP was attributed to tobacco consumption in 2014 [14]. The age-standardized prevalence of current tobacco consumption among the people with the age of ≥ 15 years was 14.9% in 2021, and it is predicted that it will decrease to 14.7% by 2030, which is not a significant achievement regarding the global targets [15, 16]. In 2016, 14.1% of Iranian adults were current users of tobacco (25.2% among men and 4.0% among women) [17]. The prevalence of tobacco use among Iranian people is rising, and it does so more quickly among women and young people [18]. Women who smoke may harm their families’ health and impose economic costs on the households. Tobacco consumption among women of reproductive ages would have adverse effects on their reproductive health, including hormone imbalance [19], irregular menstrual cycles, dysmenorrhea [20], weak reproductive function [21], and early menopause [22].

Women of reproductive ages constitute a significant proportion of the women’s population. Thus, their health decisions including tobacco consumption, would have considerable and multi-generational effects [23]. Women’s first- and second-hand tobacco consumption are among the most harmful risk factors during pregnancy. It can potentially lead to adverse pregnancy outcomes, as well as, adverse effects on the children’s health. Tobacco consumption during pregnancy significantly increases the risk of abortion [24], break of the placenta [25], premature delivery [26], stillbirth [25], low birth weight [26], neonatal infections, sudden infant death syndrome [25], and, special congenital abnormalities in the cardiovascular and digestive systems [27]. It is also associated with impaired intellectual growth [28] and learning [29].

The negative consequences of tobacco smoking go beyond the level of health and it would have adverse effects on the economic costs of households. The economic impacts of tobacco use at the individual, household, and societal levels, have been established. Low-SES households spend a larger proportion of their budget on tobacco consumption compared to those with higher SES [30]. Tobacco consumption and its inequality are a kind of complex phenomenon with multiple determinants [31, 32]. The way these inequalities occur and knowing their contributing factors can provide valuable information for developing effective interventions. Understanding the economic inequalities in tobacco consumption can help policy-making for identifying the opportunities to reduce health inequalities and resolving them. It will also allow us to comprehensively understand the socioeconomic pattern of this problem. This study was designed to investigate economic inequalities in tobacco consumption among women of reproductive ages, and its associated contributing factors using the data of the 7th Non-Communicable Disease Risk Factor Surveillance (STEPS) in Iran.

## Methods

### Source of data and the study variables

Data for this cross-sectional study was extracted from Iran’s 7th Non-Communicable Disease Risk Factor Surveillance (STEPS), conducted in 2016. A stratified-clustered random sampling was used, in which each province was considered a stratum. The sample size of each province was determined in proportion to the population size, and the sampling framework consisted of two separate lists for clusters of households living in urban and rural areas. Each cluster consisted of 10 households, whereby the total sample size was determined 31,050. More detailed information about the sampling process was presented elsewhere [33]. In this study, we used data from 10,339 women of reproductive ages (18–49 years) for the study analyses. Missing observations were insignificant and removed from the study analyses.

The outcome variables were first- and second-hand tobacco consumption. First-hand tobacco consumption refers to consuming any tobacco products through burning, chewing, inhaling, or other forms of consumption. Second-hand tobacco consumption is defined as women’s exposure to second-hand smoke at home, and in their workplace. To measure women’s economic status, an asset-based approach was applied to construct the wealth index. This index was calculated using Principal Component Analysis (PCA). In PCA, data from 30,013 individuals’ housing characteristics (residential ownership, access to piped drinking water, gas, electricity, and internet, having a bathroom, landline, kitchen, split, evaporative air cooler, and radiator), and assets (TV, LCD or LED, freezer, side-by-side refrigerator, oven-equipped stove, stove with no oven, vacuum cleaner, twin washing machine, automatic washing machine, dishwasher, personal computer, and cell phone) were used. Based on their wealth scores, individuals were classified into the 5 economic quintiles from 1st to 5th.

We utilized the categorization of Iran’s Ministry of Interior, in which provinces are divided into the five regions in terms of proximity, physical position, and similarity, to depict the geographic distribution of prevalence and inequality of tobacco consumption. The aim of classifying the provinces was to establish synergism, transfer experiences, exchange information, and regional development. Based on this classification, the northern region includes Tehran, Qazvin, Mazandaran, Semnan, Golestan, Alborz, and Qom provinces; the Central & southwest region encompasses Isfahan, Fars, Bushehr, Chahar Mahal and Bakhtiari, Hormozgan, and Kohgiluyeh and Boyer-Ahmad provinces; Northwest region includes Eastern Azerbaijan, Western Azerbaijan, Ardabil, Zanjan, Guilan, and Kurdistan provinces; Kermanshah, Ilam, Lorestan, Hamedan, Markazi, and Khuzestan were located in the West region; and Razavi Khorasan, Southern Khorasan, Northern Khorasan, Kerman, Yazd, and Sistan and Baluchistan were located in the East region.

### Measuring economic inequality

The concentration index ($C$) approach [34] was applied to measure economic inequality in first-and second-hand tobacco consumption. The index was calculated based on concentration curve which was drawn as the cumulative share of tobacco consumption on the Y-axis against the cumulative share of the women ranked by the score of economic status from the 1st to 5th quintile on the X-axis. The concentration index is calculated, as follows:

1 $$C=\frac{2}{N\mu } \sum _{i=1}^{n}{y}_{i}{r}_{i}-1$$

Where, $N$ represents the sample size, $\text{y}\text{i}$ shows the tobacco consumption of the ${i}_{th}$ woman, $\mu$ shows the mean value of tobacco consumption, and ${r}_{i}$ is the fractional rank of the ${i}_{th}$ woman in the distribution of economic status. $C$ can range from − 1 to + 1. Negative (positive) values show that tobacco consumption was more concentrated among low- (high-) economic women. There is no inequality, $C$ will be zero. Since tobacco consumption was a binary variable, the C was normalized using Wagstaff formula [35], as follows:

2 $${C}_{N}=\frac{C}{1-\mu }$$

### Decomposition of inequality

C decomposition approach [34] was applied to determine the contribution of each explanatory variable to the measured inequalities in tobacco consumption, using the formula below:

3 $${C}_{Y}=\sum _{k}(\frac{{\beta }_{k }{\stackrel{-}{X}}_{k}}{\mu }){C}_{K}+\frac{{GC}_{\epsilon }}{\mu }$$

The decomposition analysis was performed in 5 steps, as follows:

1). The estimation of the marginal effect of each explanatory variable (${\beta }_{k}$) using a logistic regression model, 2). The elasticity of each explanatory variable ($\frac{{\beta }_{k }{\stackrel{-}{X}}_{k}}{\mu }$) was calculated by multiplying the marginal effect of that variable by its mean (${\stackrel{-}{X}}_{k}$), divided by the mean value of dependent variable ($\mu$), 3). The absolute contribution of each explanatory variable to the measured inequality in tobacco consumption [$(\frac{{{\beta _k}{{\mathop X\limits^ - }_k}}}{\mu }){C_K}$] was calculated by multiplying the variable elasticity by its concentration index (${C}_{K}$), 4). $\frac{{GC}_{\epsilon }}{\mu }$ was the residual term, a part of inequality that cannot be attributable to the study’s explanatory variables, and calculated as the ${C}_{Y}$ minus the sum of absolute contributions of the explanatory variables [$\sum _{k}(\frac{{\beta }_{k }{\stackrel{-}{X}}_{k}}{\mu }){C}_{K}$], and 5). The percentage contribution of each explanatory variable and the residual term was calculated by dividing their absolute contribution by the concentration index of the outcome variable, multiplied by 100.

All of the study analyses were performed with Stata 14.

## Results

Table 1. reports descriptive statistics for women of reproductive ages in total, and by their tobacco consumption. Most of the women were middle-aged (age groups of 25–29, 30–34, and 35–39 years), and only 2.4% of women were in the age group of < 20 years. Among the study participants, 76.1% were married, 46.5% had 7–12 years of education, and 81.1% were housekeepers/unemployed/ retired. Most of the women were urban and lived in the North, Central & southwest and East regions of the country. Almost 1.1% of women drank alcohol during the last 12 months, 36.8% had stress, 36.9% had intense anger, and 28.6% had intense sadness during the last week.

The prevalence of women’s first-hand tobacco consumption was 3.6%, and it was more prevalent among those who were widowed or divorced, illiterate, self-employed, and between the ages of 45 and 49 and 35 to 39. With rising levels of economic status for women, tobacco use dropped from 5.3% among the poorest to 2.5% among the wealthiest. Furthermore, it was positively associated with drinking alcohol, stress, intense anger, intense sadness, and exposure to second-hand smoke at home and workplace (*P* < 0.05).

The prevalence of women’s exposure to home second-hand smoke was 28.3%, and it was more prevalent among women in the age groups of < 20, 40–44, and 20–24 years, and those who were married and widowed/divorced, illiterate, and manual workers. The prevalence of exposure to home second-hand smoke decreased with an improvement in women’s economic status, from 32.1% among the poorest to 18.6% among the wealthiest. Furthermore, it was more prevalent among women who drank alcohol (*P* < 0.05).

The study findings suggested that 8.4% of women were exposed to the work second-hand smoke, and women’s exposure to second-hand smoke in the workplace was more prevalent among women with the age of 40–44 years, and those who were married and widowed/divorced, illiterate, and self-employed. The prevalence of exposure to work second-hand smoke decreased with an improvement in women’s economic status, from 12% among the poorest to 5.6% among the wealthiest. It is also positively associated with drinking alcohol (*P* < 0.05).

**Table 1:** Descriptive statistics of Iranian women of reproductive ages in total and by their tobacco consumption, 2016

Women’s characteristics	Total N (%)	First-hand tobacco consumption N (%)	Exposure to home second-hand smoke N (%)	Exposure to work second-hand smoke N (%)
**All women**	10,046	358 (3.62)	2872 (28.27)	852 (8.38)
**Age**				
< 20	231 (2.35)	2 (0.93)	76 (32.44)	20 (8.79)
20–24	1274 (12.87)	29 (2.23)	381 (30.08)	116 (8.52)
25–29	1792 (17.68)	54 (3.17)	497 (27.39)	146 (8.49)
30–34	1960 (19.69)	67 (3.40)	542 (27.09)	166 (8.47)
35–39	1736 (17.41)	75 (4.20)	492 (27.93)	140 (7.81)
40–44	1520 (14.89)	56 (3.87)	474 (30.84)	141 (9.03)
45–49	1533 (15.10)	75 (5.15)	410 (26.52)	123 (7.98)
***P*** ***-value*** ^*^		***< 0.001***	***0.048***	***0.928***
**Marital status**				
Single	1861 (18.90)	43 (2.36)	451 (23.80)	134 (7.20)
Married	7682 (76.07)	269 (3.56)	2282 (29.42)	674 (8.67)
Widowed/divorced	503 (5.02)	46 (9.41)	139 (27.70)	44 (8.47)
***P-value***		***< 0.001***	***< 0.001***	***0.150***
**Education**				
Illiterate	606 (5.82)	50 (8.37)	258 (42.37)	92 (14.26)
1–6	2472 (23.98)	123 (5.18)	930 (37.51)	270 (11.01)
7–12	4596 (46.49)	136 (3.01)	1273 (27.68)	357 (7.76)
> 12	2372 (23.71)	49 (2.10)	411 (16.63)	133 (5.50)
***P-value***		***< 0.001***	***< 0.001***	***< 0.001***
**Job**				
Housekeeper/unemployed/retired	8176 (81.06)	300 (3.73)	2460 (29.84)	708 (8.58)
Office worker	663 (6.72)	12 (1.85)	94 (13.89)	39 (6.00)
Manual worker	138 (1.40)	7 (5.35)	45 (33.18)	13 (10.69)
Self-employed	454 (4.60)	27 (6.18)	135 (29.14)	58 (12.31)
Student	615 (6.21)	12 (1.88)	138 (21.65)	34 (4.96)
***P-value***		***0.001***	***< 0.001***	***< 0.001***
**Economic status**				
1st quintile (Poorest)	1884 (18.65)	99 (5.25)	608 (32.06)	223 (12.00)
2nd quintile	1941 (19.11)	81 (4.34)	689 (34.95)	195 (9.61)
3rd quintile	2044 (20.18)	69 (3.31)	651 (31.74)	169 (8.06)
4th quintile	2088 (20.88)	58 (2.92)	535 (25.27)	149 (7.12)
5th quintile (Wealthiest)	2089 (21.17)	51 (2.54)	389 (18.55)	116 (5.64)
***P-value***		***< 0.001***	***< 0.001***	***< 0.001***
**Area of residence**				
Rural	2976 (28.98)	143 (4.84)	1138 (37.46)	398 (13.01)
Urban	7070 (71.02)	215 (3.13)	1734 (24.52)	454 (6.49)
***P-value***		***< 0.001***	***< 0.001***	***< 0.001***
**Region**				
North	2670 (28.11)	64 (2.48)	519 (19.41)	147 (5.47)
Central & southwest	2040 (19.75)	133 (6.62)	757 (36.47)	199 (9.38)
Northwest	1738 (17.38)	21 (1.26)	562 (31.71)	187 (10.11)
West	1583 (16.23)	21 (1.41)	509 (32.47)	159 (9.93)
East	2015 (18.52)	119 (6.34)	525 (26.07)	160 (8.74)
***P-value***		***< 0.001***	***< 0.001***	***< 0.001***
**Drinking alcohol**				
No	9941(98.92)	329 (3.35)	2812 (27.97)	829 (8.24)
Yes	105 (1.08)	29 (28.92)	60 (55.89)	23 (21.02)
***P-value***		***< 0.001***	***< 0.001***	***< 0.001***
**Stress**				
No	6358 (63.17)	181 (2.89)	-	-
Yes	3688 (36.83)	177 (4.89)	-	-
***P-value***		***< 0.001***	-	-
**Intense anger**				
No	6319 (63.11)	162 (2.62)	-	-
Yes	3727 (36.89)	196 (5.34)	-	-
***P-value***		***< 0.001***	-	-
**Intense sadness**				
No	7179 (71.45)	208 (2.92)	-	-
Yes	2867 (28.55)	150 (5.38)	-	-
***P-value***		***< 0.001***	-	-
**Exposure to home second-hand smoke**				
No	7174 (71.73)	89 (1.26)	-	-
Yes	2872 (28.27)	269 (9.63)	-	-
***P-value***		***< 0.001***	-	-
**Exposure to work second-hand smoke**				
No	9194 (91.62)	274 (3.01)	-	-
Yes	852 (8.38)	84 (10.40)	-	-
***P-value***		***< 0.001***	-	-

- : Not included in the analysis, because no relationship was hypothesized between the covariate and the outcome variable

^*^ Chi-square test *P*-values

Based on the findings in Table 2, the prevalence of women’s first-hand tobacco consumption was higher in rural than urban areas (4.8% vs. 3.1%). The greatest frequency was reported by women in the Central & southwest and East regions, at 6.6% and 6.3%, respectively. Rural areas had a higher prevalence of exposure to the second-hand smoke at home than urban areas (37.5% vs. 24.5%). The Central & southwest (36.5%), West (32.5%), and Northwest (31.7%) regions had the greatest prevalence. Women from rural areas reported more exposure to work second-hand smoke than their urban counterparts (13% vs. 6.5%). Northwest, West, and Central & southwest regions had the highest prevalence of 10.1%, 9.9%, and 9.4%, respectively.

Regarding the concentration indices in Table 2, first-hand tobacco consumption was more concentrated among low-economic women than their high-economic counterparts in the whole country, rural areas, and Central & southwest and East regions. There were no significant economic inequalities in first-hand tobacco consumption in urban areas and other regions. Furthermore, there was a pro-rich inequality in women’s home second-hand smoke in the whole country, urban areas, and all of the regions (it was more concentrated among low-economic women). In rural areas, however, home second-hand smoke was distributed equally among women from different economic strata. The inequality gap in work second-hand smoke was also pro-rich in the country, urban areas, and Central & southwest, Northwest, and West regions.

**Fig. 1:**
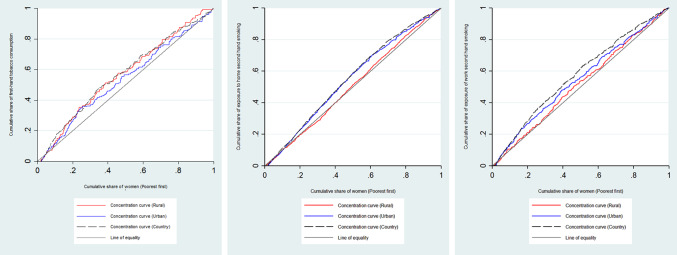
The concentration curves of first- and second-hand tobacco consumption among Iranian women of reproductive ages, 2016

**Fig. 2:**
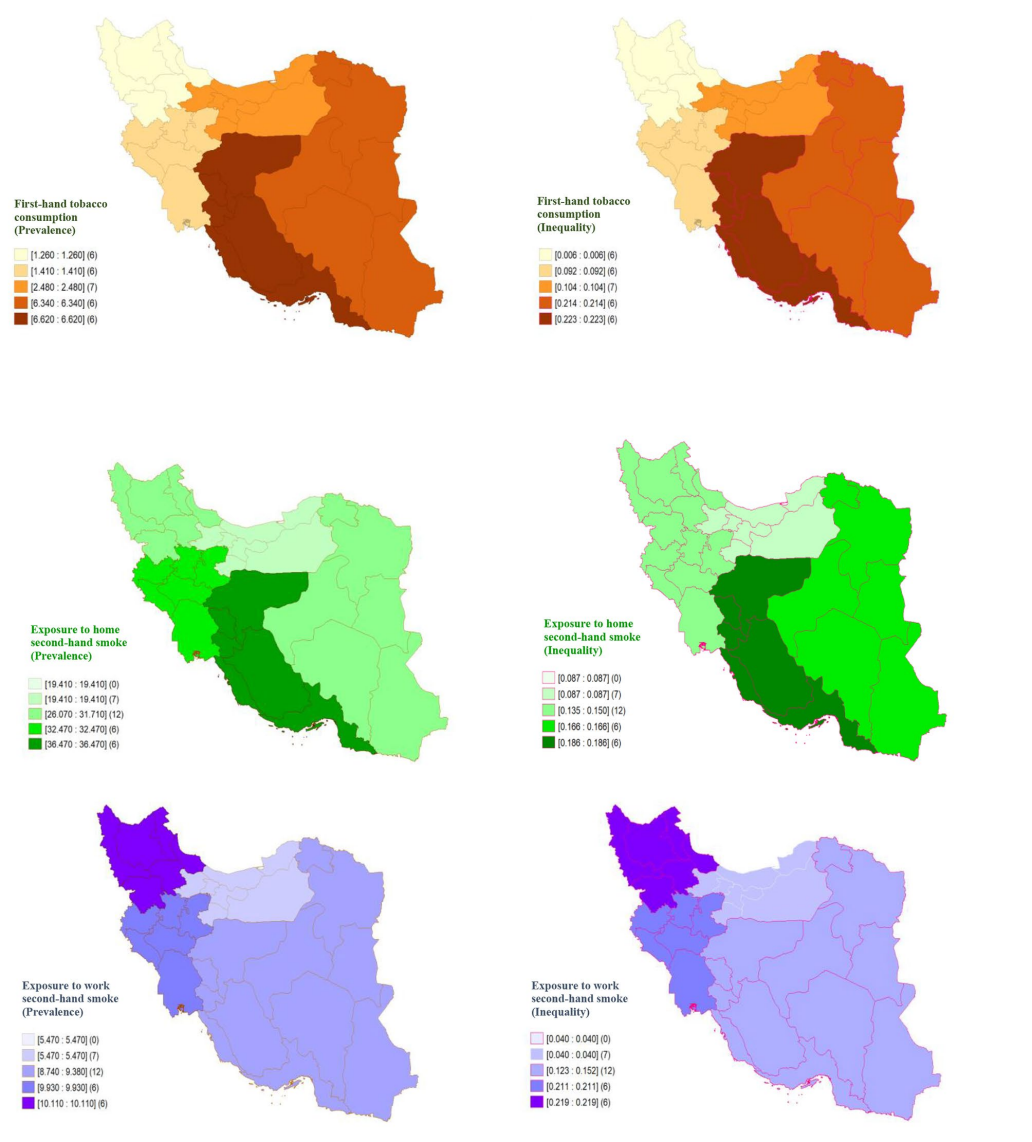
Regional distribution of prevalence and economic inequalities of first- and second-hand tobacco consumption among Iranian women of reproductive ages, 2016

Figure 1. shows the concentration curves of the first- and second-hand tobacco consumption in the country and rural and urban areas, in which lying the concentration curves above the line of equality, and crossing the line of equality suggests the pro-rich and equal distribution of the study outcomes, respectively. Furthermore, the regional distribution of the prevalence and economic inequality of first- and second-hand tobacco consumption is illustrated in Fig. 2.

**Table 2:** Geographical distribution of prevalence and economic inequality of first- and second-hand tobacco consumption among Iranian women of reproductive ages, 2016

Variables	N (n)^*^	Prevalence (95% CI^*^)	Concentration index (95% CI^*^)	*P*-value
**First-hand tobacco consumption**				
Country	10,046 (358)	3.62 (3.18, 4.07)	-0.141 (-0.179, -0.104)	< 0.001
Rural areas	2976 (143)	4.84 (3.83, 5.86)	-0.140 (-0.121, -0.032)	0.018
Urban areas	7070 (215)	3.13 (2.66, 3.60)	-0.077 (-0.200, -0.081)	0.082
*P*-value	-	*< 0.001*	*0.390*	-
Regions				
North	2670 (64)	2.48 (1.86, 3.10)	0.104 (0.027, 0.182)	0.180
Central & southwest	2040 (133)	6.62 (5.34, 7.89)	-0.223 (-0.281, -0.165)	< 0.001
Northwest	1738 (21)	1.26 (0.71, 1.80)	0.006 (-0.123, 0.136)	0.960
West	1583 (21)	1.41 (0.76, 2.06)	0.092 (-0.032, 0.217)	0.459
East	2015 (119)	6.34 (4.89, 7.80)	-0.214 (-0.280, -0.148)	< 0.001
*P*-value	-	*< 0.001*	*0.008*	-
**Exposure to home second-hand smoke**				
Country	10,046 (2872)	28.27 (27.07, 29.47)	-0.151 (-0.167, -0.135)	< 0.001
Rural areas	2976 (1138)	37.46 (35, 39.92)	-0.021 (-0.146, -0.108)	0.458
Urban areas	7070 (1734)	24.52 (23.18, 25.87)	-0.127 (-0.050, 0.007)	< 0.001
*P*-value	-	*< 0.001*	*0.002*	-
Regions				
North	2670 (519)	19.41 (17.53, 21.29)	-0.087 (-0.118, -0.056)	< 0.001
Central & southwest	2040 (757)	36.47 (33.55, 39.38)	-0.186 (-0.219, -0.154)	< 0.001
Northwest	1738 (562)	31.71 (28.72, 34.71)	-0.150 (-0.186, -0.113)	< 0.001
West	1583 (509)	32.47 (29.39, 35.55)	-0.135 (-0.173, -0.098)	< 0.001
East	2015 (525)	26.07 (23.38, 28.76)	-0.166 (-0.206, -0.127)	< 0.001
*P*-value	-	***< 0.001***	***0.137***	-
**Exposure to work second-hand smoke**				
Country	10,046 (852)	8.38 (7.64, 9.12)	-0.162 (-0.189, -0.135)	< 0.001
Rural areas	2976 (398)	13.01 (11.24, 14.77)	-0.040 (-0.135, -0.068)	0.323
Urban areas	7070 (454)	6.49 (5.74, 7.24)	-0.102 (-0.080, 0.001)	< 0.001
*P*-value	-	*< 0.001*	*0.237*	-
Regions				
North	2670 (147)	5.47 (4.38, 6.56)	-0.040 (-0.102, 0.022)	0.522
Central & southwest	2040 (199)	9.38 (7.56, 11.21)	-0.152 (-0.207, -0.096)	< 0.001
Northwest	1738 (187)	10.11 (8.22, 12)	-0.219 (-0.281, -0.157)	< 0.001
West	1583 (159)	9.93 (7.98, 11.89)	-0.211 (-0.271, -0.151)	< 0.001
East	2015 (160)	8.74 (6.90, 10.58)	-0.123 (-0.187, -0.059)	0.053
*P*-value	-	*< 0.001*	*0.022*	-

^*^N: Women interviewed, n: The number of women who were for example, current tobacco users in the whole country and in urban and rural areas, CI: Confidence Interval

Based on the results of decomposition analyses in Table 3, exposure to home second-hand smoke, education, and economic status had the largest contributions to the measured inequality in women’s first-hand tobacco consumption (48.9%, 38.9%, and 30.8%, respectively). Education, economic status, and residency in rural areas were the three main factors that explained 43.8%, 30.3%, and 18% of the measured inequality in women’s exposure to home second-hand smoke. Similarly, 42.2%, 38.8%, and 32% of the measured inequality in women’s exposure to work second-hand smoke was attributable to residency in rural areas, economic status, and education, respectively.

**Table 3:** Decomposing economic inequalities in first- and second-hand tobacco consumption among Iranian women of reproductive ages, 2016

Women’s characteristics	First-hand tobacco consumption						Exposure to home second-hand smoke						Exposure to work second-hand smoke					
	Marginal effect	Elasticity	C_k_	Absolute contribution	Contribution (%)	Summed contribution (%)	Marginal effect	Elasticity	C_k_	Absolute contribution	Contribution (%)	Summed contribution (%)	Marginal effect	Elasticity	C_k_	Absolute contribution	Contribution (%)	Summed contribution (%)
Age (RC*: <20)																		
20–24	0.031	0.111	-0.035	-0.004	2.73	**-4.52**	-0.014	-0.006	-0.035	0.219*10^− 3^	-0.15	**1.94**	-0.006	-0.009	-0.035	0.300*10^− 3^	-0.19	**1.57**
25–29	0.044	0.217	-0.031	-0.007	4.78		-0.046	-0.029	-0.031	0.001	-0.59		-0.009	-0.019	-0.031	0.001	-0.36	
30–34	0.045	0.249	-0.014	-0.003	2.39		-0.064	-0.044	-0.014	0.001	-0.40		-0.013	-0.030	-0.014	0.401*10^− 3^	-0.25	
35–39	0.051	0.245	0.013	0.003	-2.19		-0.071	-0.044	0.013	-0.548*10^− 3^	0.36		-0.024	-0.050	0.013	-0.001	0.39	
40–44	0.044	0.184	0.023	0.004	-2.95		-0.066	-0.035	0.023	-0.001	0.52		-0.017	-0.030	0.023	-0.001	0.42	
45–49	0.056	0.234	0.056	0.013	-9.27		-0.111	-0.059	0.056	-0.003	2.19		-0.025	-0.045	0.056	-0.003	1.55	
**Marital status (RC: Single)**																		
Married	-0.001	-0.023	0.038	-0.001	0.63	**3.50**	0.052	0.139	0.038	0.005	-3.48	**-3.21**	0.015	0.137	0.038	0.005	-3.19	**-3.04**
Widowed & divorced	0.024	0.034	-0.120	-0.004	2.87		0.019	0.003	-0.120	-0.398* *10^− 3^	0.26		0.003	0.002	-0.120	-0.252*10^− 3^	0.16	
**Education (RC: Illiterate)**																		
1–6 years	-0.014	-0.092	-0.298	0.027	-19.38	**38.86**	-0.042	-0.035	-0.298	0.011	-6.98	**43.83**	-0.015	-0.042	-0.298	0.013	-7.74	**32.01**
7–12 years	-0.024	-0.305	0.068	-0.021	14.81		-0.117	-0.193	0.068	-0.013	8.76		-0.032	-0.179	0.068	-0.012	7.56	
> 12 years	-0.026	-0.170	0.361	-0.061	43.42		-0.209	-0.176	0.361	-0.063	42.04		-0.051	-0.145	0.361	-0.052	32.18	
**Job (RC: Housekeeper/unemployed/retired)**																		
Office worker	-0.012	-0.023	0.428	-0.010	7.00	**5.40**	-0.060	-0.014	0.428	-0.006	4.06	**3.75**	0.011	0.009	0.428	0.004	-2.36	**-1.93**
Manual worker	0.004	0.002	-0.009	-0.013*10^− 3^	0.01		0.082	0.004	-0.009	-0.036*10^− 3^	0.02		0.036	0.006	-0.009	-0.053*10^− 3^	0.03	
Self-employed	0.010	0.013	0.070	0.001	-0.64		0.023	0.003	0.070	0.267*10^− 3^	-0.18		0.043	0.024	0.070	0.002	-1.02	
Student	0.004	0.007	0.208	0.001	-0.97		0.005	0.001	0.208	0.238*10^− 3^	-0.16		-0.015	-0.011	0.208	-0.002	1.42	
**Economic status (RC: 1st quintile: Poorest)**																		
2nd quintile	-0.002	-0.012	-0.539	0.006	-4.60	**30.84**	0.042	0.028	-0.539	-0.015	10.08	**30.27**	-0.010	-0.022	-0.539	0.012	-7.48	**38.75**
3rd quintile	-0.009	-0.051	-0.054	0.003	-1.92		0.032	0.023	-0.054	-0.001	0.81		-0.016	-0.039	-0.054	0.002	-1.30	
4th quintile	-0.005	-0.031	0.465	-0.015	10.36		-0.005	-0.004	0.465	-0.002	1.24		-0.018	-0.044	0.465	-0.020	12.60	
5th quintile (Wealthiest)	-0.007	-0.038	1.000	-0.038	27.00		-0.037	-0.027	1.000	-0.027	18.13		-0.022	-0.057	1.000	-0.057	34.93	
**Area of residence (RC: Urban)**																		
Rural	0.002	0.017	-0.521	-0.009	6.14	**6.14**	0.051	0.052	-0.520	-0.027	18.02	**18.02**	0.038	0.131	-0.521	-0.068	42.24	**42.24**
**Region (RC: East)**																		
North	-0.027	-0.207	0.193	-0.040	28.28	**8.21**	-0.035	-0.034	0.193	-0.007	4.39	**5.13**	-0.021	-0.069	0.193	-0.013	8.21	**9.69**
Central & southwest	-0.005	-0.029	0.042	-0.001	0.86		0.114	0.080	0.042	0.003	-2.23		0.015	0.034	0.042	0.001	-0.89	
Northwest	-0.062	-0.298	-0.003	0.001	-0.65		0.049	0.030	-0.003	-0.093*10^− 3^	0.06		0.015	0.031	-0.003	-0.096*10^− 3^	0.06	
West	-0.055	-0.245	-0.117	0.029	-20.27		0.065	0.037	-0.117	-0.004	2.90		0.017	0.032	-0.117	-0.004	2.32	
**Drink alcohol (RC: No)**																		
Yes	0.071	0.021	0.303	0.006	-4.52	**-4.52**	0.302	0.011	0.303	0.003	-2.31	**-2.31**	0.101	0.013	0.303	0.004	-2.42	**-2.42**
**Stress (RC: NO)**																		
Yes	0.002	0.020	0.056	0.001	-0.80	**-0.80**	-	-	-	-	-	-	-	-	-	-	-	-
**Intense anger (RC: No)**																		
Yes	0.012	0.125	0.022	0.003	-1.97	**-1.97**	-	-	-	-	-	-	-	-	-	-	-	-
**Intense sadness (RC: No)**																		
Yes	0.002	0.012	-0.021	-0.254*10^− 3^	0.18	**0.18**	-	-	-	-	-	-	-	-	-	-	-	-
**Exposure to home second hand smoke (RC: No)**																		
Yes	0.059	0.458	-0.151	-0.069	48.88	**48.88**	-	-	-	-	-	-	-	-	-	-	-	-
**Exposure to work second hand smoke (RC: No)**																		
Yes	0.011	0.026	-0.162	-0.004	3.04	**3.04**	-	-	-	-	-	-	-	-	-	-	-	-
**Total observed**				**-0.188**		**133.24**				**-0.147**		**97.42**				**-0.189**		**116.86**
**Residual**				**0.047**		**-33.24**				**-0.004**		**2.48**				**0.027**		**-16.78**
**Total**				**-0.141**		**100**				**-0.151**		**100**				**-0.162**		**100**

^*^ Reference category

- : Not included in the analysis, because no relationship was hypothesized between the covariate and the outcome variable.

## Discussion

This study investigated economic inequalities in first- and second-hand tobacco consumption among women of reproductive ages in Iran. We also assessed economic inequalities in women’s tobacco consumption in rural and urban areas and five geographic regions of the country. The findings of this study are interpreted in two sections, as follows

### Economic inequalities in tobacco consumption in the country

The results indicated that the prevalence of tobacco consumption among women of reproductive age was 3.6%. The prevalence of women’s tobacco smoking in Iran was more than that of Armenia (1.6%) and Egypt (0.5%), and less than its prevalence in Turkey (19.7%) and Bahrain (5.7%). Tobacco consumption is less prevalent among women than men in Iran and this is true for other countries [17]. This could indicate a kind of social stigma for women according to which, they are less expected to smoke [36]. Additionally, social norms may influence the types of tobacco products women use. Cigarette smoking is not viewed as a respectable behavior among women, but the use of smokeless tobacco products in India or hookah in Iran is more prevalent among women and young girls than the use of other tobacco products [37, 38]. This is because these products do not conflict with social norms as much as smoking does. We found there is a pro-rich inequality of women’s first-hand tobacco consumption, suggesting that it was more concentrated among low-economic women. The findings of decomposition analysis indicated exposure to home second-hand smoke, education, and economic status, were the main contributors to the measured inequality in women’s tobacco consumption.

In line with our results, other studies showed exposure to second-hand smoke at home is one of the risk factors for tobacco consumption [39, 40]. Our results show that low-economic women are more exposed to second-hand smoke at home than the high-economic women. As shown in other studies, female smokers are more prohibited by their non-smoker partners from smoking [41]. Moreover, access to tobacco products is positively associated with young women’s smoking initiation and adverse transition in smoking over time [39]. It seems that having at least a family member who uses tobacco could change the norms of low-economic families in line with women’s tobacco consumption and increase their access to tobacco products.

Education and economic status were the next contributors to the measured inequality in women’s tobacco consumption. Studies confirmed that high-risk lifestyles, such as tobacco consumption tend to be more prevalent among poor [32, 42–45] and low-educated women [42, 45]. Smoking uptake may be more prevalent and attempts to quit may be less prevalent among low-SES individuals who consider tobacco consumption as a mechanism for coping with the stressful living environment [46]. Moreover, there are fewer social pressures not to smoke for smokers with low socioeconomic positions [47]. In a variety of contexts, lower economic status is associated with reduced access to care, poorer health outcomes [48], and less health literacy [49]. According to studies, social support, motivation to quit, addiction to tobacco, treatment adherence, psychological differences like low self-efficacy, and marketing by the tobacco industry are some of the factors that could account for variations in tobacco consumption and quitting between high- and low-economic individuals [46]. The prevalence of exposure to home and work second-hand smoke was 28.2% and 8.4%, respectively. Less exposure to second-hand smoke in the workplace could be in terms of the law that bans smoking in public places, including all workplaces [10, 11]. However, as Alimohammadi et al. in their study report, “there is a huge gap between ratified laws, and performing of laws” [50]. Results of a study in Iran showed that exposure to second-hand smoke is not much different between Iranian men and women, while women are more exposed to second-hand smoke at home, men are more exposed to second-hand smoke in the workplace [17]. The findings of our research also indicated pro-rich inequalities in women’s exposure to secondhand smoke at home and at workplace, suggesting that low-economic women experienced negative effects of secondhand smoke more severely. Level of education, economic status, and residency in rural areas had the largest positive contributions to the measured inequalities in women’s exposure to home and work second-hand smoke. These findings are consistent with previous research which found a positive relationship between women’s low SES, including income, education, and residency in rural areas, and their exposure to second-hand smoke [51–54]. Studies also suggested that low-SES women are more likely to be exposed to second-hand smoke in their workplace [55, 56]. Low-SES people do not have much awareness and have less negative attitudes towards the risks of second-hand smoke [57, 58], and may not be able to control or change environmental conditions and complain about improving them. More prevalence of first-hand smoking among low-SES households [31] could be another reason for more exposure of their female members to second-hand smoke. Moreover, it appears that less-educated women are less able to acquire relevant information and are less familiar with how to seek for it on various social media [57]. Greater exposure to secondhand smoke among rural women compared to urban women may be a consequence of demographic differences such as lower income, education, and employment status, but it may also reflect sociocultural differences [59]. This may be due to the high rates of unemployed women in rural areas compared to their urban counterparts. Based on the results of a study on Bangladesh culture, most women are housewives, especially in rural areas, and spend most of their time at home, which increases the risk of exposure to second-hand smoke from the smoking men at home [52].

### Geographical distribution of economic inequalities in tobacco consumption

Findings about the geographical distribution of inequalities in tobacco consumption revealed diverse patterns across rural and urban areas and the five regions of the country.

Women’s first-hand tobacco consumption was more prevalent in rural areas, and poor women suffered from more first-hand tobacco consumption in this setting. The low population density in rural areas leads to decreased access to healthcare services, and communication, causing low-SES people with inadequate health literacy [60] to be unaware of the adverse consequences of tobacco consumption [61]. Furthermore, there may be a lack of tobacco control policies and other supervisory structures in rural areas [62]. Meanwhile, rural areas are less likely to have a workplace, thus tobacco production provides employment opportunities in these areas [63], such that it becomes normalized further in the culture, and the social context in which low-SES people exert less opposition to smoking [47]. Poor households are more vulnerable regarding tobacco consumption and its associated economic consequences, aggravating, and sustaining their vulnerable situation in rural areas [64].

The highest prevalence of women’s tobacco consumption was reported in Central & southwest and East regions, which can be in terms of greater access to tobacco products. Moreover, adjacency to neighboring countries, and having racial, cultural, and behavioral similarities with Iraqi Kurds and the people of Pakistan and Afghanistan could be a reason for the high prevalence of women’s tobacco consumption in these two regions [17]. Our results showed that tobacco consumption in these two regions is significantly more concentrated among low-economic women. This finding can be justified by the fact that the provinces located in these two regions are in a wide range of development levels due to human development index (HDI) [65]. It seems that improving the level of economic and social development in less-developed provinces and increasing women’s health literacy could reduce the prevalence, and economic inequalities in high-risk lifestyles, including tobacco consumption.

Exposure to home second-hand smoke was more prevalent among rural than urban women, and it was more concentrated among low-economic women in urban areas. This suggests that home smoking rules are less perceived and followed by family members in rural than the urban settings and among low-economic households in urban areas [66]. It may be due to a dearth of education and awareness regarding the negative effects of secondhand smoke exposure [63]. In addition, rural and low-economic urban households may have a larger number of members and fewer rooms to implement smoking home rules. In addition, women who live in these households are more likely to consider tobacco smoking as the right of men in their families [17]. These differences could somehow have resulted from differences in social context [47].

The study findings showed that the prevalence of exposure to second-hand smoke in workplaces was higher among rural women compared to their urban counterparts, and it was more concentrated among low-economic women in urban areas. This is consistence with the results of a study that suggested smoking rules in workplaces are less perceived and followed by people in rural areas than the urban settings and exposure to the work second-hand smoke is more prevalent among rural residents [66]. In our study, the higher prevalence of exposure to second-hand smoke at work among rural than urban women could be because in the villages, most of the women do their economic activities at home, where family members may not be as strict about smoking rules as workmates outside the family. The study’s results show that despite the implementation of the restricting tobacco smoking rules in some workplaces in urban areas, low-economic women still work in environments where these regulations are either not enforced or enforced incompletely.

Women’s exposure to second-hand smoke at home and workplace was more prevalent in Central & southwest, Northwest, and Western regions of the country, which could be explained by the more prevalence of smoking among men in some provinces of these regions [17]. In all regions, there was a pro-rich inequality in women’s exposure to home second-hand smoke. In all regions, except for the East and the North, low-economic women were more likely to be exposed to secondhand smoke in the workplace. Additionally, women from these two regions reported less exposure to secondhand smoke at home and in the workplace. Based on HDI, the provinces located in these two regions are not significantly different from their neighboring provinces in terms of the level of development. Provinces of North region (Tehran, Alborz, Mazandaran, and Semnan) were at a very high level of HDI, and in the East region, most of the provinces are at an average level of HDI [65]. Therefore, balanced development in these two regions can be the reason for the equal distribution of exposure to work second-hand smoke among low- and high-economic women. These findings reveal the need for interventions to establish and enforce restricting smoking rules in families and workplaces, especially in less developed provinces.

Despite anti-tobacco measures in Iran, there are still economic inequalities in women’s tobacco consumption and exposure to second-hand smoke. Narrowing these inequality gaps requires more government efforts to design and implement gender-based policies and women-oriented measures. Creating women-oriented anti-tobacco campaigns, especially for low-SES groups, in less-developed geographic regions and at all workplaces, as well as, removing the cultural and financial barriers to access to tobacco quitting services, could provide more opportunities to support low-SES women to stop smoking and their less exposure to second-hand smoke. In order to design gender-sensitive initiatives, monitoring and surveillance data on tobacco consumption and exposure to secondhand smoke must be disaggregated by gender [37]. To the best of our knowledge, this is the first study in Iran that assessed economic inequalities in first- and second-hand tobacco consumption among Iranian women. In this study, the data from a national survey was used, which increased the generalizability of the research results due to the large sample size. However, this study was subject to some limitations; First, we had no data on women’s income, therefore, an asset-based index was used to measure women’s economic status. Second, because of the cross-sectional design of the study, causal interpretations should be done with caution.

## Conclusion

The results of the present study could shed light on the most important driving factors of inequalities in women’s tobacco consumption, and provide the primary evidence for future planning and adoption of the right policies. The study indicated a diversity in the geographical distribution of the inequalities in rural and urban areas, and five regions of the country. These findings highlighted the need for more enforcement of tobacco control rules and increasing tobacco taxes as general measures. Furthermore, there is a need for gender-sensitive initiatives at the national and regional levels to educate, support, and empower low-SES women and households for tobacco cessation and complying with restrictive smoking rules.

## Data Availability

The data that support the findings of this study are available from Iran’s National Institute of Health. The study data also are available from the corresponding authors upon reasonable request and with permission of the National Institute of Health Research.
